# Special Teaching Formats during the COVID-19 Pandemic—A Survey with Implications for a Crisis-Proof Education

**DOI:** 10.3390/jcm10215099

**Published:** 2021-10-30

**Authors:** Karin Christine Huth, Leonard von Bronk, Maximilian Kollmuss, Stefanie Lindner, Jürgen Durner, Reinhard Hickel, Miriam Esther Draenert

**Affiliations:** Department of Conservative Dentistry and Periodontology, University Hospital, LMU Munich, Goethestrasse 70, 80336 Munich, Germany; lvonbron@dent.med.uni-muenchen.de (L.v.B.); kollmuss@dent.med.uni-muenchen.de (M.K.); stlindne@dent.med.uni-muenchen.de (S.L.); juergen.durner@med.uni-muenchen.de (J.D.); hickel@dent.med.uni-muenchen.de (R.H.); mdraener@dent.med.uni-muenchen.de (M.E.D.)

**Keywords:** dental education, COVID-19, OSPE, communications skills, remote

## Abstract

Modern teaching formats have not been considered necessary during the COVID-19 pandemic with uncertain acceptance by students. The study’s aim was to describe and evaluate all measures undertaken for theoretical and practical knowledge/skill transfer, which included objective structured practical examinations (OSPEs) covering a communication skills training. The students’ performance in the OSPE as well as the theoretical knowledge level were assessed, of which the latter was compared with previous terms. In conservative dentistry and periodontology (4th and 5th year courses), theoretical teaching formats were provided online and completed by a multiple-choice test. Practical education continued without patients in small groups using the phantom-head, 3D printed teeth, and objective structured practical examinations (OSPEs) including communication skills training. Formats were evaluated by a questionnaire. The organization was rated as very good/good (88.6%), besides poor Internet connection (22.8%) and Zoom^®^ (14.2%) causing problems. Lectures with audio were best approved (1.48), followed by practical videos (1.54), live stream lectures (1.81), treatment checklists (1.81), and virtual problem-based learning (2.1). Lectures such as .pdf files without audio, articles, or scripts were rated worse (2.15–2.30). Phantom-heads were considered the best substitute for patient treatment (59.5%), while additional methodical efforts for more realistic settings led to increased appraisal. However, students performed significantly worse in the multiple-choice test compared to the previous terms (*p* < 0.0001) and the OSPEs revealed deficits in the students’ communication skills. In the future, permanent available lectures with audio and efforts toward realistic treatment settings in the case of suspended patient treatment will be pursued.

## 1. Introduction

On 12 March 2020, the World Health Organization announced that the Coronavirus disease-19 (COVID-19) was considered as a global pandemic [1], affecting all areas of life around the world. This includes all fields of dentistry [2,3], from dental care [4] to education. Dentists are known to have a high risk of infection due to possible transmission via aerosols and droplets [5,6], which are unavoidable for many dental procedures as well as close proximity to many patients. As a result, increased hygienic demands and requirements for social distancing were implemented within the already high hygiene standards in dentistry [7]. Dental students face the same potential risks as dental health care staff within the patients’ treatment and chairside education. In particular, chairside dental education creates a high proximity between the patient, the student with assistance, and the teacher. Therefore, students and professionals across the globe [8,9,10,11] were faced with the challenge of adapting dental education in a crisis-proof manner [12]. The current literature stresses the demand for an international exchange of measures to address this new challenge [13]. Furthermore, a huge portfolio of different teaching approaches has been described. The scope ranges from recorded lectures, live stream lectures, and online conferences using various platforms (e.g., Microsoft Teams, Blackboard, or Zoom) [11,14,15,16] to final examinations in the form of MEQs (modified essay questions), MCQs (multiple choice questions), OSCEs, and online interviews [17]. Most formats have been compared to face-to-face teaching, mainly in terms of student’s attitude rather than objective assessment of knowledge level. During the first lockdown in early 2020, and in part ongoing throughout 2020 and 2021, measures have been undertaken to minimize the risk of infection for the students, the staff, and the patients [18,19]. Different measures have been reported internationally to reduce the contact between staff and patients, most importantly, personal protection equipment (PPE), entrance checkpoints with temperature measurements, and contact history questionnaires as well as workforce shift schedules [20]. Aside from the increased hygienic measures, the education of undergraduate dental students was switched from face-to-face education to online lecturing and practical teaching without patients. To compensate for the resulting lack of patient communication, a new OSPE (objective structured practical examination) was additionally performed and evaluated. 

The objective structured practical examination (OSPE) is an examination format primarily in medical school that is designed to assess clinical competence. This examination format consists of a course with different stations in which practical skills, theoretical knowledge, and communication skills are tested [21,22]. Problem-based learning (PBL) is an educational learning model in which a clinical problem serves as an impetus for active learning. Participants work together in small groups to define their own learning objectives and gain a comprehensive understanding of a problem. Here, the aim of PBL is to develop strategies to solve a complex dental patient case. It also helps to develop clinical reasoning, teamwork, and communication skills [23].

The aim of this study was to describe all measures and special formats of theoretical and practical knowledge/skills transfer within the undergraduate clinical dental education in the field of conservative dentistry and periodontology during the COVID-19 summer term in 2020. Furthermore, the students’ attitude toward this alternative teaching concept was surveyed by an electronic questionnaire. In addition, the theoretical knowledge level was assessed and compared with previous terms. We explored the following questions: Is teaching without practical patient contact as efficient as teaching with patients? Do additional digital teaching formats support knowledge transfer? In light of the present literature, the added value of this study may derive from issuing not only a theoretical, but also practical and communication skills training without patients such as a training and assessment OSPE in periodontology followed by structured feedback.

From the results, conclusions can be drawn, of which the alternative education formats should be maintained in the future for a contemporary, crisis-proof, and sustainable dental education.

## 2. Materials and Methods

### 2.1. Setting and Participants

The special teaching formats and their evaluation involved 4th and 5th year undergraduate students (*n* = 86) attending the first and second clinical course in the Department of Conservative Dentistry and Periodontology, Ludwig-Maximilians University, Munich, from April until August 2020 (approval of the ethics committee no. 20-547 KB, 15.06.2020). The participants (69% female, 31% male; mean age 26.5 years) were motivated to participate in the evaluation after completing the OSPE. The questionnaire contained different parts: (1) General (two questions); (2) Digital setting (five questions), (3) Theoretical teaching (20 questions); and (4) Practical teaching (10 questions). The 5th year students also evaluated the OSPE (eight questions). The questions regarding teaching formats were rated as either very good, good, satisfactory, bad, and very bad. In terms of using the Likert scale, we considered the answer choices of fully agree, rather agree, partially agree, disagree, and fully disagree. A detailed explanation of the increased hygienic measures for clinical as well as educational settings at our department can be found in Diegritz et al. [7]. Furthermore, separate entrances for students were installed, two groups were formed to diminish the number of students simultaneously present in the building, and one-way walking markings were set up in the course rooms as well as distance lines in waiting areas (e.g., in front of the students’ stock issue).

### 2.2. Theoretical Education

Within a regular term, the face-to-face lectures are available afterward as .pdf files on Moodle^®^ (Modular Object-Oriented Dynamic Learning Environment, West Perth WA 6872, Australia) together with scripts, checklists, videos, and virtual problem-based learning seminars (VHB). 

In the Corona summer term of 2020, the theoretical education began with a systematic six-week theory module, while the practical education was postponed to avoid any social contact. The majority of lectures were either prerecorded with an audio file or broadcast as a live-stream with online chat and recorded, which were both available on Moodle^®^ throughout the entire term. Most of them were also available as .pdf files. Six additional videos regarding organizational procedures, hygiene briefing, and rules of conduct were generated, while videos showing specific treatment sequences were increased up to 64. Table 1 shows the change in the theoretical education elements from the regular semester to the 2020 Corona summer term in detail.

In pediatric dentistry, the new digital competences of staff and students were valued to offer an international webinar on thee “oral pathology in children” by an Australian specialist via Zoom^®^ (Zoom video communications, San Jose, CA 95113, USA). 

### 2.3. Practical Education

Normally, students are trained in practical and communication skills by treating patients supervised by Assistant Professors according to the National Competency Based Catalogue of Dental Education (NKLZ) [24]. In the 2020 Corona summer term, the educational challenge was to create, even without patients, an add on in practical skills training and clinical context compared with the phantom course in the first clinical term. Next to a realistic phantom head positioning without the possibility of tooth replacement accompanied by realistic hygiene procedures, each field has found its own approach to accomplish this task. In the field of restorative dentistry, the fabrication of ceramic partial crowns and inlays (4th year) and veneers (5th year) is required. In endodontology, students of both courses receive problem-based learning (PBL) [21,23] seminars followed by realistic and comparative training in root canal treatment using 3D printed teeth based on patient DVT data [25]. 

In periodontology, the 5th year students solely received practical training at the phantom head, while the 4th year students first ran through a PBL tutorial. Each student received an exemplary patient case, which should be planned from anamnesis and diagnosis up to prognosis and treatment planning. They had to virtually present and discuss their cases in small supervised groups via Zoom^®^. In the following, an individual objective structured practical examination (OSPE) [22,26] was implemented covering practical skills (34 achievable points) and theoretical knowledge (34 points) as well as communication skills training (32 points). The latter was accomplished by a fellow student acting as a patient based on a prewritten script regarding anamnesis facts and oral health complaints together with respective X-rays, while the assessment was based on the Calgary Cambridge Observation Guide (CCOG) [27]. The OSPE was followed by structured feedback [22,28], and upon agreement, the student could watch the recorded session to ensure self-reflection and competence development [29,30].

Regarding the difference between the 4th and 5th year courses, the 5th year students had to undertake 33.3% more practical work than the 4th year students, while the education methods were the same for both courses except in periodontology.

### 2.4. Final Examination

At the end of the 2020 summer term, theoretical examinations took place for both courses, each consisting of 30 “Pick N Type” multiple-choice (MC) questions with four answer choices [31,32]. The results were compared to the mean of the grades of the four previous terms (2018 summer term up to the 2019/2020 winter term).

### 2.5. Questionnaire 

A questionnaire (Table 2) was developed and electronically provided via Moodle^®^, which anonymously evaluated the students’ acceptance regarding organization, technical requirements, and the different theoretical and practical teaching formats. It was addressed through the student’s identification number for both the 4th and 5th year students, whereby the 4th year questionnaire contained additional questions about the OSPE in periodontology.

In addition to site-specific questions, literature research was conducted with the search terms e-learning, remote learning, students’ acceptance of dental education, evaluation/assessment of new teaching forms in dentistry, COVID-19 dental education, and digitalization within the dental curriculum, which resulted in a pool of 143 questions after the elimination of redundancies. Based on the nominal group technique (NGT) [33,34], a prioritization of the questions was carried out by the authors assigning 1–3 points to each question according to its subjective importance, yielding a descending list according to the sum of points awarded. To ensure a feasible time to complete the questionnaire of around 15 min, the list was cut from the end to a total of 62 questions followed by a content related arrangement. The answer options were mostly based on modified Likert scales [35,36,37], while multiple answer options or free text were also included.

### 2.6. Statistics 

Data were analyzed descriptively and given graphically (Microsoft Excel, version 16.43, Redmond, WA, USA; GraphPad Prism, San Diego, CA, USA). Theoretical examination scores, separately for the 4th and 5th year courses, were statistically compared (Mann–Whitney U-test) between groups (2020summer term versus pooled previous four terms) after testing the normal distribution of the data (Shapiro–Wilk test). Alpha level was set at ≤0.05. The power as well as the effect size *d* were also given for the comparisons (GPower 3.1).

## 3. Results

The response rate of the questionnaire was 91% (4th year 42/45 students, 5th year 37/42 students, total 79/87, female 55 (69.6%), male 24 (30.4%)).

### 3.1. Evaluation of the General Learning Conditions 

The organization was rated as “very good” (44.3%) or “good” (44.3%) (together 88.6%) by the majority of students (satisfactory 10.1%, bad 1.3%, very bad 0%). While most participants did not experience any technical problems (63.3%), the most frequent complaints were a poor Internet connection (22.8%) and problems with Zoom^®^ (14.2%). 

### 3.2. Evaluation of Theoretical Teaching Formats 

**Figure 1:**
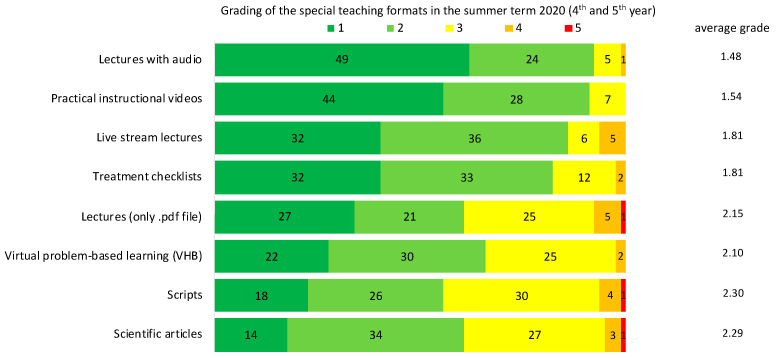
Students’ (*n* = 79 out of 86) overall assessment of different teaching formats in grades. Evaluation was conducted using school grades: 1 = very good, 2 = good, 3 = satisfactory, 4 = bad, 5 = very bad. Given are the different teaching formats (left), the absolute number of different grades per teaching form (middle), and the average grade (right).

Using grades from 1 (very good) to 5 (poor), lectures with audio were rated best with an average grade of 1.48 (Figure 1), followed by videos (1.54), live stream lectures including the webinar in pediatric dentistry (1.81), treatment checklists (1.81), and virtual problem-based learning (VHB) (2.1). Lectures only available as .pdf files (2.15), scientific articles (2.29), and scripts (2.30) were at the end. The aforementioned pediatric webinar (38 participating students) was particularly highlighted among the free-text responses, due to the promoting of English terminology, and the interaction with an international lecturer.

**Figure 2:**
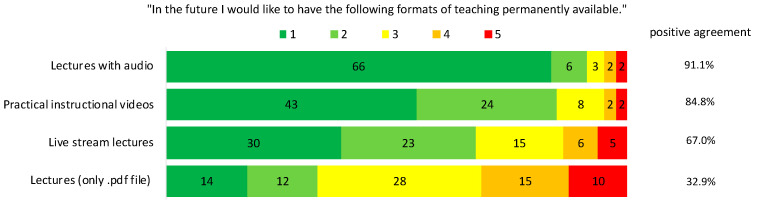
Evaluation of different teaching formats regarding the students’ wish for maintenance. Rating was conducted using a modified Likert scale, 1 = fully agree, 2 = rather agree, 3 = partially agree, 4 = rather disagree, 5 = disagree at all. Given are the newly developed teaching formats (left), and the absolute number of given answers (middle). In addition, ratings 1 and 2 are given together as positive agreement (right).

Regarding the students’ wish to maintain the newly developed theoretical teaching formats in the future, lectures with audio were mostly named (91% “fully agree” and “partially agree”), followed by the videos (85%), live stream lectures (67%), and lectures provided solely as .pdf files at the end (32.91%) (Figure 2).

**Figure 3:**
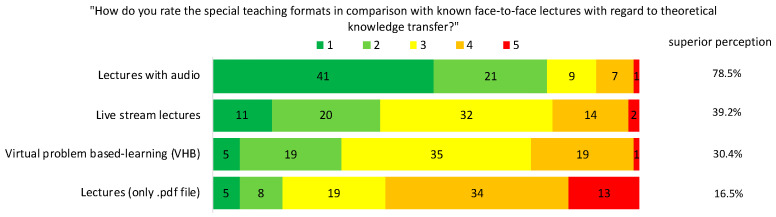
Assessment of the virtual theoretical teaching formats in comparison with traditional theoretical face-to-face teaching. Evaluation was conducted using a modified Likert scale, 1 = distinctly better, 2 = better, 3 = similar, 4 = worse, 5 = distinctly worse. Given are the different theoretical teaching formats (left), and the absolute number of given ratings (middle). In addition, ratings 1 and 2.

Furthermore, the students were asked to compare the virtual theoretical teaching formats with the traditional face-to-face teaching regarding their efficacy of knowledge transfer (Figure 3). The ratings “distinctly better” and “better” were taken together and considered as “superior perception”. This was the case in 78.5% for the lectures with audio, 39.2% for the live stream lectures, 30.4% for the virtual problem-based learning (VHB), and only 16.5% for the lectures solely given as .pdf files.

### 3.3. Evaluation of the Practical Training

Most students fully or rather agreed that the phantom head was the best possible replacement for treatment of a patient (59.5%). However, 69.4% of the students indicated, that they did not have the same respect toward the phantom head. Only 30.4% of the students stated they would appreciate additional training opportunities at the phantom head in the future. As advantages, the students primarily mentioned a relaxed working atmosphere (64.6%), equal conditions for all students (55.7%), gaining practical routine by working without complications (no saliva, no tongue; 54.4%), and no risk of infection (54.4%). As disadvantages, the lack of specific treatment parts, for example, injection (96.2%), anatomical individuality (96.2%), patient feedback such as pain perception (93.7%), and lack of communication (87.3%) were named.

The practical teaching in restorative dentistry was rated as “very good” or “good” by 85.7% of the 4th year and 81.0% of the 5th year students. In endodontology, it was rated as “very good” or “good” by 83.3% (4th year) and even by 91.1% of the 5th year students. In periodontology, it was graded as “very good” or “good” by 78.6% (4th year students), however, by only 27.0% of the 5th year students. The 4th year students rated the combination of tutorial and OSPE to be more realistic due to the simulated communication (69.0%), and appreciated it as further training of the complex periodontal diagnostic process, and would welcome its maintenance in the future (71.4%). Analyzing the students’ performance within the OSPE, they achieved 79.8 ± 9.6% (mean ± SD) of the possible points (theoretical part 77.8 ± 3.6%, practical part 91.9 ±3.2%, communication skills 67.2 ± 1.0%).

Taking the theoretical and practical education together, 95% of the students rated their progress in theoretical knowledge and practical skills equal to that in a regular term.

### 3.4. Final Examination

**Figure 4:**
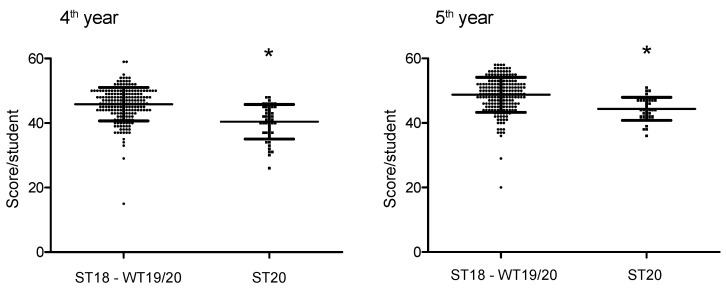
Evaluation of final examinations under regular and Corona conditions. Given are the 4th (**left**) and 5th year (**right**) students’ scores in the final theoretical examination; each graph contrasts the results from the 2018 summer term to the 2019/2020 winter term (ST18-WT19/20), the results of the 2020 Corona summer term (ST20) (each dot represents one student, the bars depict mean ± SD, Mann–Whitney U-Test, * *p* < 0.0001).

In the 2020 summer term, the 4th year students achieved 40.4 ± 5.3 points, and the 5th year students 44.4 ± 3.6 points (mean ± SD) (maximum score 60 points, pass mark 36 points). This was significantly less than in the pooled examinations of the last four terms (4th year 45.9 ± 5.2 points, and 5th year 48.8 ± 5.4 points (mean ± SD) (*p* < 0.0001) (Figure 4). The comparison within the 4th year and 5th year students before and during Corona showed a power of 99.98 and an effect size d of 1.04 as well as 99.88 and 0.95, respectively.

## 4. Discussion

### 4.1. Theoretical Education

Regarding theoretical education, several approaches toward online availability were developed such as lectures with audio, live stream lectures, practical instructional videos, and introducing videos, which replaced hygienic or equipment briefings on site. The newly acquired online skills were also used for international exchange (webinar in pediatric dentistry). According to the questionnaire, lectures with audio were rated best among these approaches and should be maintained in the future, according to the opinion of most of the students. They especially emphasized the availability at any time and place via the Internet, and the possibility to repeat and pause sequences. These features would create a highly efficient and individual learning, which can be adapted to their own pace and daily routines. Interestingly, formats such as scripts, scientific articles, and lectures given solely as .pdf files without audio were rated worse, which may underline the importance of embedding content into an explaining frame. Besides, although not counted in particular, lecturers had the feeling of a higher number of attendees during their online lectures than their past face-to-face lectures. In contrast, it became clear from the free text comments within the survey that the students missed the communication between each other and the staff to eliminate uncertainties. Furthermore, they disapproved the lack of a clear separation of life and work, which would have a negative impact on their learning motivation. The mentioned findings are in accordance with recent studies from all over the world (e.g., Germany, Italy, and Jordan), which also evaluated the online learning possibilities during the COVID-19 pandemic [11,15,16,38,39].

However, the presented study revealed no hints that students would feel insufficiently qualified for patient treatment after the 2020 summer term, as this was critically mentioned by various studies [11,39] nor did the presented study point to an increased burnout risk, which was brought up as a concern in the case of sustained teaching without attendance [40,41]. In contrast, 95% of our students rated their progress in theoretical knowledge and practical skills equal to that in a regular term, although their theoretical test results were worse compared to the previous terms. Most of the recent studies assessed the students’ subjective attitude toward the different teaching formats rather than their effectiveness in terms of knowledge gain. Instead, we tried to objectively evaluate their knowledge level based on end of term examinations. In this way, the success of the corona term in its entirety was evaluated and not solely the effect of specific formats.

### 4.2. Practical Education

To substitute the suspended patient treatment in the 2020 summer term, several approaches were offered to fulfill the practical education in this crisis situation, which ranged from demanding preparations at the phantom head, PBL presentations in combination with root canal treatments in 3D-printed teeth [25] to tutorials and OSPEs including communication skills training with acting patients. Although the students’ treatment using phantom heads were only a surrogate for real patient treatment, the participants highly appreciated the provided efforts. However, this positive response should be viewed critically, since the newly offered formats could appear subjectively interesting and tended to be evaluated more positively. As disadvantages, the lack of typical treatment attributes such as salivation, tongue movements, patient feedback, anatomical individuality, and communication were named.

Interestingly, 4th year students rated the education in periodontology better than the 5th year students. The difference was that the 4th year course was provided with a tutorial and an OSPE including communication training with an acting patient in addition to the practical skills training at the phantom head. The 5th year course trained solely at the phantom head. Therefore, one could conclude that the more patient-centered simulation of periodontal treatment covering different aspects such as in this case, anamnesis, diagnosis, and treatment planning, taking the information of a simulated patient into account in combination with communication, found more acceptance by the students. Another aspect might have been, that the 5th year students had already treated patients in their 4th year course and therefore felt the difference to the phantom head and the associated loss of the mentioned attributes possibly even more. Communication was deemed important as a key process within the patient–doctor relationship as stressed already in the literature [27,42,43,44]. This is consistent with well-known learning theories that emphasize the need for teaching methods with social interaction. This is not only evident with a view to Miller’s pyramid of competencies [45], but also according to Kolb [46], who, following the concept of “experiential learning”, describes learning as a social process supported by experiences, educational moments, and a safe environment. In the 2020 summer term, the OSPE format was not primarily used for assessment but rather an excellent teaching tool seeking a higher level of competency [26]. According to Miller´s pyramid of competence [45], we could reach level 3a “shows how” (i.e., performance based on simulated patients). This requires the knowledge regarding cognitive levels 1 “know” and 2 “knows how”, which was taught in the tutorials and at the phantom head.

As a welcome side effect when looking at the free text comments, the participants highlighted the better supervision relation between students and staff. Interestingly, the new approbation regulation for dentists, which becomes effective as of 2021, will provide a better relation of 1:3 for treatments of patients and of 1:6 for teaching at the patient [47].

## 5. Conclusions

Regarding the theoretical education, the study especially suggests maintaining digital lectures with audio on demand in future education accompanied by Q&A sessions with staff. A stable Internet connection is a prerequisite of utmost importance. While nothing can really replace patient treatment in practical education, for a crisis proof practical training/assessment without patients, approaches toward a higher level of competence such as OSPEs should be applied including as many aspects of patient treatment as possible. Special attention should also be paid to the training of communication skills. Nevertheless, a limitation of this study is that only students from a single dental school participated in this study. Therefore, especially, in view of the high relevance of this subject, review articles are necessary to gain a comprehensive overview of the measures undertaken worldwide.

## Figures and Tables

**Table 1:** Theoretical and practical dental education in the 2020 Corona summer term in comparison to a regular term (4th and 5th year courses, Department of Conservative Dentistry and Periodontology).

	Teaching Format	Regular Term	Corona Summer Term 2020
Theoretical Education	Lectures with audio (*n*)	None	41
			33 additionally as .pdf files
			8 exclusively as lectures with audio
	Live stream lectures (*n*)	None	10
			8 additionally as .pdf files
			2 exclusively as live stream lectures
	Practical instructional videos (*n*)	62	64
	Introducing videos (e.g., hygiene briefing) (*n*)	None	6
	Treatment checklists (*n*)	7	7
	Lectures (only .pdf file) (*n*)	41	43
			33 additionally as lectures with audio
			10 exclusively available as .pdf files)
	Virtual problem-based learning (VHB) (*n*)	6	6
	Scripts (*n*)	2	2
	Scientific articles/recommended literature (*n*)	None	6
	Case presentation (endodontology) (*n*)	None	1
	Tutorial periodontology (*n*)	None	1
Practical education	Restorative Dentistry	Regular patient treatment	Phantom head treatment,
			add on in clinical difficulty (veneers)
	Endodontology	Regular patient treatment	Phantom head treatment,
			Problem based Learning (PBL) case presentations, root canal treatment using 3D printed teeth
	Periodontology	Regular patient treatment	Phantom head treatment,
			Tutorial (PBL) followed by virtual presentations OSPE including communication training with simulated patients

**Table 2:** Two exemplary questions regarding the evaluation of the different teaching formats in the summer term 2020 together with answer options.

How do you rate your personal learning success with regard to the individual theoretical teaching forms in the summer term 2020?					
Lectures with audio	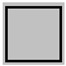 Very good	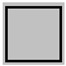 Good	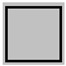 Satisfactory	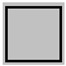 Bad	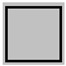 Very bad
Live Stream lectures	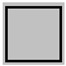 Very good	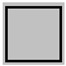 Good	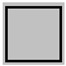 Satisfactory	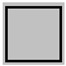 Bad	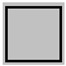 Very bad
Lectures (only .pdf files)	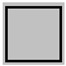 Very good	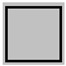 Good	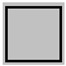 Satisfactory	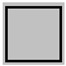 Bad	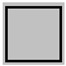 Very bad
Scripts	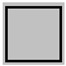 Very good	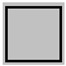 Good	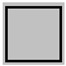 Satisfactory	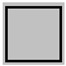 Bad	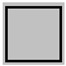 Very bad
Scientific articles	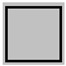 Very good	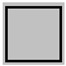 Good	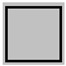 Satisfactory	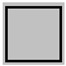 Bad	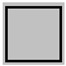 Very bad
Virtual problem-based learning (VHB)	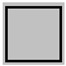 Very good	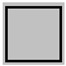 Good	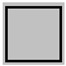 Satisfactory	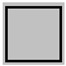 Bad	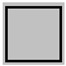 Very bad
In the future I would like to have “Lectures with audio” permanently available	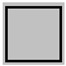 Fully agree	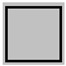 Rather agree	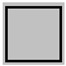 Partially agree	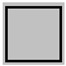 Disagree	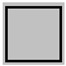 Disagree at all

## Data Availability

The data can be made available upon reasonable request from the corresponding author.
